# Autonomic Nervous System Dysfunction in Pediatric Sepsis

**DOI:** 10.3389/fped.2018.00280

**Published:** 2018-10-09

**Authors:** Colleen M. Badke, Lauren E. Marsillio, Debra E. Weese-Mayer, L. Nelson Sanchez-Pinto

**Affiliations:** ^1^Division of Critical Care Medicine, Ann & Robert H. Lurie Children's Hospital of Chicago, Chicago, IL, United States; ^2^Department of Pediatrics, Northwestern University Feinberg School of Medicine, Chicago, IL, United States; ^3^Center for Autonomic Medicine in Pediatrics, Ann & Robert H. Lurie Children's Hospital of Chicago, Chicago, IL, United States; ^4^Stanley Manne Children's Research Institute, Chicago, IL, United States

**Keywords:** autonomic nervous system, pediatrics, sepsis, organ dysfunction, critical care, heart rate variability, inflammation

## Abstract

The autonomic nervous system (ANS) plays a major role in maintaining homeostasis through key adaptive responses to stress, including severe infections and sepsis. The ANS-mediated processes most relevant during sepsis include regulation of cardiac output and vascular tone, control of breathing and airway resistance, inflammation and immune modulation, gastrointestinal motility and digestion, and regulation of body temperature. ANS dysfunction (ANSD) represents an imbalanced or maladaptive response to injury and is prevalent in pediatric sepsis. Most of the evidence on ANSD comes from studies of heart rate variability, which is a marker of ANS function and is inversely correlated with organ dysfunction and mortality. In addition, there is evidence that other measures of ANSD, such as respiratory rate variability, skin thermoregulation, and baroreflex and chemoreflex sensitivity, are associated with outcomes in critical illness. The relevance of understanding ANSD in the context of pediatric sepsis stems from the fact that it might play an important role in the pathophysiology of sepsis, is associated with outcomes, and can be measured continuously and noninvasively. Here we review the physiology and dysfunction of the ANS during critical illness, discuss methods for measuring ANS function in the intensive care unit, and review the diagnostic, prognostic, and therapeutic value of understanding ANSD in pediatric sepsis.

## Introduction

**Figure 1:**
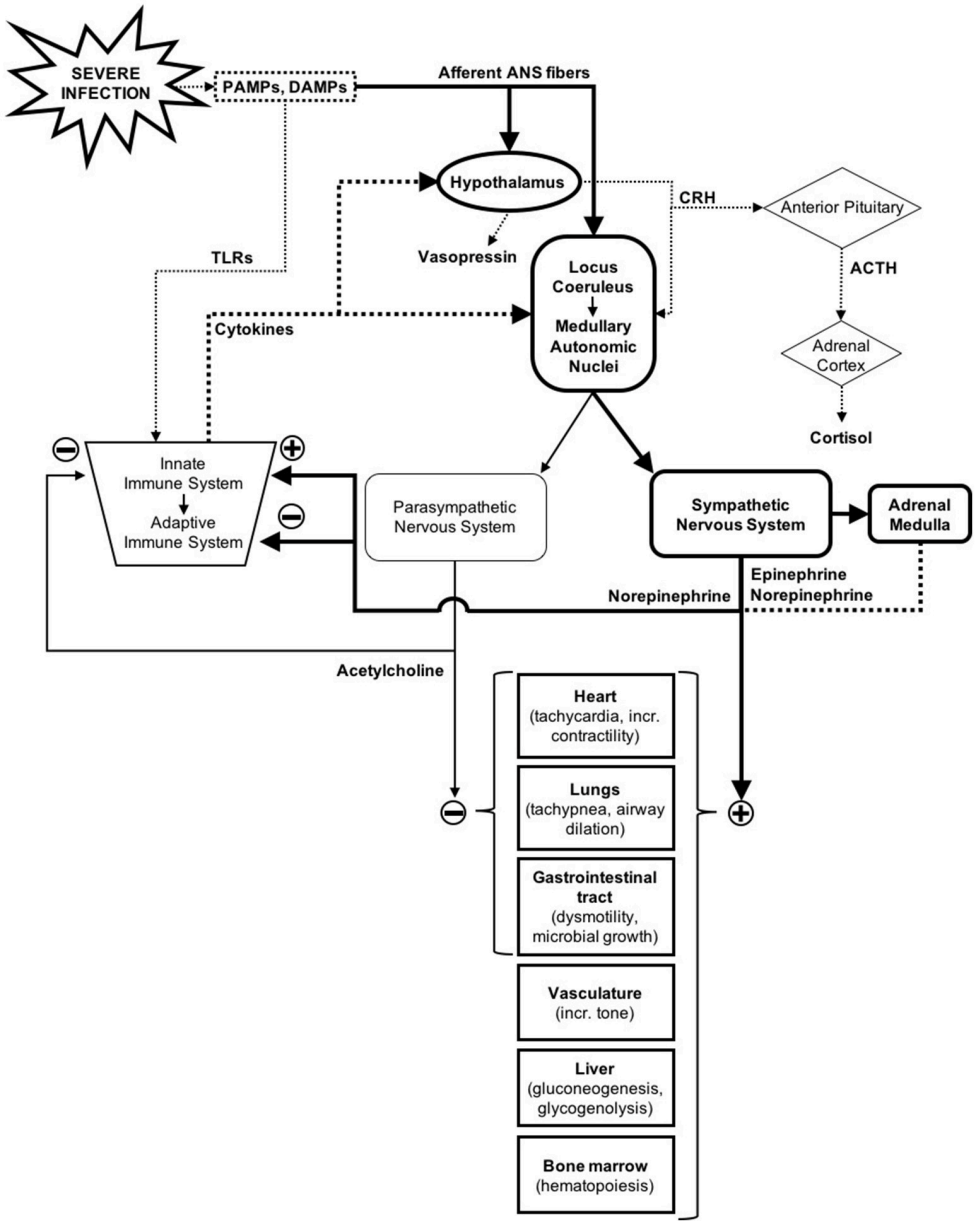
Autonomic nervous system response to severe infections in the context of the immune and neuroendocrine response. Solid lines represent nerve pathways and dotted lines represent humoral/endocrine pathways (2–4).

The autonomic nervous system (ANS) is a unique system as it regulates functions in nearly all organ systems (1). Along with immune and neuroendocrine responses, the ANS plays a major role in maintaining homeostasis when confronted with internal and external stressors, including severe infections (Figure 1) (2–4). The homeostatic processes in which the ANS has a primary role is extensive, but some of the most relevant during sepsis include regulation of cardiac output, vascular tone, control of breathing, airway resistance, regulation of the inflammatory response, adaptive immune modulation, gastrointestinal motility, and thermoregulation (1, 5–8).

In response to severe infections and other injuries, the central nervous system (CNS) activates the sympathetic branch of the ANS in order to make the necessary physiological and metabolic adjustments to overcome the acute physiologic stress (4). ANS dysfunction (ANSD) represents an imbalanced or maladaptive response to stress, and is often due to excessive, uncontrolled, or prolonged sympathetic activation, or inappropriate regulation by the other branch of the ANS, the parasympathetic nervous system (3). Most of the evidence on ANSD in critical care stems from research on heart rate variability (HRV), which is a marker of ANS function that is inversely correlated with organ dysfunction and mortality in both adult and pediatric patients (9–13). However, there is evidence that other markers of ANSD such as respiratory rate variability, skin thermoregulation, and baroreflex and chemoreflex sensitivity are also associated with outcomes in critically ill adults (14–16).

There are several reasons why understanding ANSD in the context of sepsis is crucial to the care of the critically ill septic child. First, sepsis is defined as a “life-threatening organ dysfunction caused by a dysregulated host response to infection” (17). Although “host response” is often equated to “immune response” in the literature, there is evidence that a dysregulated ANS response to infection is common in patients with sepsis and an early marker of organ dysfunction (18). If indeed ANSD is a central component of sepsis pathophysiology, this could have major implications in the development of new diagnostic and therapeutic strategies for sepsis. In addition, several surrogates of ANSD, including HRV and respiratory rate variability (RRV), are associated with organ dysfunction, treatment response, and outcomes in neonates, children, and adults and can be measured continuously and noninvasively (19–23). Furthermore, changes in some of these surrogates of ANSD can be detected up to 18 h before the onset of shock, which can make them effective early predictors of clinical deterioration (24).

In this paper we will review the physiology and dysfunction of the ANS during critical illness, discuss methods for measuring ANS function in the pediatric intensive care unit, and review the diagnostic and prognostic value of ANSD in pediatric sepsis.

## Autonomic nervous system physiology and dysfunction in sepsis and critical illness

### Overview

The ANS is responsible for regulating all innervated organs (except the skeletal muscles), a remarkable task often overlooked by clinicians (1, 25, 26). It has direct effects on cardiovascular function, immunity, gastrointestinal function, thermoregulation, and other key adaptive mechanisms to stress (1, 5–8) The autonomic efferent fibers are functionally and anatomically divided into the sympathetic and parasympathetic nervous systems. Both systems can work antagonistically (e.g., in the heart, airways, and gastrointestinal tract), independently (e.g., sympathetic-mediated vascular tone), or synergistically (e.g., visual accommodation) (26). Most of the efferent response is achieved through smooth muscle constriction and relaxation, glandular secretion, cardiac muscle constriction, and cardiac cell conductivity regulation (1).

The *sympathetic nervous system* (SNS) is often characterized as the system in charge of the “fight or flight” response; however, it also carries important roles in day-to-day, non-danger situations (1, 27). Post-ganglionic sympathetic nerves travel across most of the body and secrete norepinephrine to activate the adrenergic receptors in their target organs and tissues, except in the kidney, where they secrete dopamine (Table 1). Chromaffin cells in the adrenal medulla also secrete catecholamines (about ~80% epinephrine and ~20% norepinephrine) into the circulation, which then travel throughout the body to activate adrenergic receptors. Additionally, the chromaffin cells secrete enkephalins which bind to opioid receptors to produce an analgesic effect (1).

**Table 1:** Sympathetic Adrenergic and Parasympathetic Muscarinic Receptors: Response to Activation and Drug Effects.

**Sympathetic adrenergic receptor**	**Response to activation**	**Drug effects**
α_1_	• Arterial and arteriolar vasoconstriction • Pupillary dilation • Decreased gastrointestinal motility and sphincter contraction • Hepatic glycogenolysis and gluconeogenesis • Pro-inflammatory cytokine production • Central nervous systems effects including anorexia	**Receptor Activation**: • Norepinephrine, epinephrine (α_1_,α_2_) • Phenylephrine, midodrine (α_1_) • Clonidine, dexmedetomidine (α_2_)
α_2_	• Decreased norepinephrine release through autoreceptors (negative feedback) • Decreased acetylcholine release through parasympathetic heteroreceptors • Central nervous systems effects including sedation, analgesia, and down-regulation of sympathetic outflow (= hypotension, bradycardia)	**Receptor Blockade**: • Phentolamine, Phenoxybenzamine (α_1_, α_2_) • Doxazosin (α_1_)
	• Decreased gastrointestinal motility and gland secretion • Decreased insulin secretion • Coronary, renal, and skin vasoconstriction • Constriction of veins • Platelet aggregation • Monocyte-endothelial adhesion	**Norepinephrine Reuptake Inhibition**: • Cocaine, methylphenidate, amphetamines, tricyclic antidepressants.
**ß**_1_	• Increased heart rate, cardiac conductivity, and cardiac muscle contractility • Renin release from the kidney	**Receptor Activation**: • Norepinephrine, epinephrine (β_1_, β_2_) • Isoproterenol (β_1_, β_2_) • Dobutamine (β_1_) • Albuterol, terbutaline (β_2_)
**ß**_2_	• Bronchodilation • Decreased bronchial gland secretion • Potent coronary vasodilatation (exceeds α_2_ constriction effect)	
	• Increased heart rate, cardiac conductivity, and cardiac muscle contractility • Bladder relaxation • Skeletal muscle, pulmonary, and visceral vasodilation • Immune modulation in lymphoid tissue	**Receptor Blockade**: • Propranolol (β_1_, β_2_) • Atenolol, esmolol, metoprolol, nadolol, timolol (β_1_ > β_2_)
**ß**_3_	• Lypolysis and thermogenesis in adipose tissue	**Norepinephrine Reuptake Inhibition**: • Cocaine, methylphenidate, amphetamines, tricyclic antidepressants.
**Parasympathetic muscarinic receptor**	**Response to activation**	**Drug effects**
M_1_	• Gastric acid secretion • Pancreatic amylase secretion • Cerebral vasoconstrictio	**Receptor activation**: • Acetylcholine (M_1_-M_4_) • Methacholine (M_1_-M_4_)
M_2_, M_3_	• Decreased heart rate, decreased cardiac conductivity • Pupillary and ciliary constriction • Lacrimal, salivary, nasopharyngeal, bronchial, and digestive gland secretion • Bronchial constriction • Increased gastrointestinal motility • Sphincter relaxation	**Receptor Blockade**: • Atropine, ipratropium, scopolamine (M_1_-M_4_) • Oxybutynin (M_3_)
M_2_, M_4_	• Decreased acetylcholine release through autoreceptors (negative feedback) • Decreased norepinephrine release through sympathetic heteroreceptors	**Inhibition of Acetylcholinesterase**: • *Reversible*: Edrophonium, neostigmine, physostigmine, pyridostigmine • *Irreversible*: Organophosphates

*α_1_ and ß_3_ are activated by norepinephrine > epinephrine; α_2_ and ß_1_ are activated by norepinephrine = epinephrine; ß_2_ are activated by epinephrine >> norepinephrine. Muscarinic receptors in the target organs (and the nicotinic receptors in the post-ganglionic neurons) are activated by acetylcholine (1)*.

The *parasympathetic nervous system* (PNS) is often referred to as the “rest and digest” system, and is in charge of energy conservation, digestion, and waste removal. The vagus nerve, which innervates most organs and tissues between the larynx and the small intestine, controls the vast majority of parasympathetic functions in the body (1, 25). Post-ganglionic parasympathetic nerves secrete acetylcholine to activate the muscarinic receptors in their target organs and tissues (Table 1), except in the immune system where they primarily activate nicotinic receptors (1, 5).

### Cardiovascular and respiratory regulation

Most cardiovascular effects are mediated by the SNS, and their effects can be readily appreciated in the extremes of sympathetic dysfunction (loss of function and over-activity). Loss of function is best exemplified by spinal cord injuries, where the lack of sympathetic tone leads to orthostatic hypotension, low resting blood pressure, loss of circadian blood pressure fluctuation, and bradycardia (28). Sympathetic over-activity, seen in states of stress including critical illness, can lead to tachyarrhythmias, vasoplegia due to downregulation of adrenergic receptors, cardiac ischemia, and heart failure (3, 27, 29).

In homeostasis, heart function is tightly controlled by sympathetic activation of the ß-adrenergic receptors and parasympathetic activation of the muscarinic M_2_ receptors. Sympathetic input increases SA node-mediated heart rate (chronotropy), AV node and ventricular conductivity (dromotropy), and atrial and ventricular contractility (inotropy). Parasympathetic input through the vagus nerve has a negative effect on all of these cardiac functions, except ventricular contraction, which is unaffected (1).

The baroreceptor reflex mediates the synergistic control of blood pressure by both efferent branches of the ANS. Increases in blood pressure lead to baroreceptor activation in the carotid sinuses and aortic arch, which leads to a decrease in sympathetic tone and increase in vagal tone that result in a drop in the heart rate and blood pressure. When the blood pressure is too low, a decrease in baroreceptor activity leads to the opposite effect: increased vasoconstriction, tachycardia, and cardiac contractility leading to a blood pressure increase (1, 30).

Cardio-respiratory homeostasis is primarily modulated through the chemoreceptor reflex, which is in part responsible for RRV (31). A decrease in arterial oxygen content, a rise in carbon dioxide, or a drop in pH sensed peripherally and centrally by chemoreceptors results in an increase in respiratory rate, depth of breathing, and heart rate. Both baroreceptor and chemoreceptor sensitivity can be altered in critical illness-induced ANSD and this dysregulation is thought to be a risk factor for organ dysfunction (30).

### Immune and inflammatory response

The influence of the ANS on the immune system is complex but extremely important, with effects on both the innate and the adaptive immune response (Figure 1) (7). Whereas humoral and cellular modulation may take hours or days to take effect, the response of the ANS can be instantaneous, inviting the hypothesis that the ANS may play a significant role in the early-phase immune response during sepsis (5).

The best-described ANS-immune system interaction occurs through the cholinergic anti-inflammatory pathway, which modulates the innate immune system response. Initially the CNS senses inflammation through both the humoral route (mediated by cytokines) and the neural route [mediated by the ANS afferent sensory fibers, which can sense damage- and pathogen-associated molecular patterns [DAMPs and PAMPs]] (2, 5). This leads to increased PNS output that results in acetylcholine release by the vagus nerve in the reticuloendothelial system (spleen, liver, thymus, etc.). Acetylcholine activates the nicotinic receptors of tissue macrophages, which leads to a decrease in secretion of pro-inflammatory cytokines like IL-6 and TNF-α (5, 7). The importance of the vagus nerve integrity in this pathway has been established in experimental murine models, which have shown that direct electrical stimulation of the vagus nerve results in decreased TNF-α secretion, whereas vagotomy results in the opposite effect (5). Increased PNS activity, measured using HRV, has been associated with reduced pro-inflammatory cytokine levels, suggesting that the cholinergic anti-inflammatory pathway could be monitored non-invasively using HRV (32).

The SNS has effects on both the adaptive and the innate immune systems. Epinephrine and norepinephrine activation of α_1_-adrenergic receptors in reticuloendothelial macrophages can lead to pro-inflammatory cytokine release, although in prolonged septic states, epinephrine down-regulates IL-6 and TNF-α production (33). In parallel, norepinephrine activation of ß_2_-adrenergic receptors in helper T cells in the lymphatic tissue can lead to an immunosuppressive Th2 polarization, particularly in the setting of sepsis (7, 33).

### Neuroendocrine response

Neuroendocrine physiology is closely linked to autonomic regulation (Figure 1) (1). In response to perturbation, like inflammation and tissue damage in sepsis, the CNS activates both ANS and neuroendocrine pathways (i.e., the hypothalamic-pituitary-adrenal [HPA] axis, the hypothalamic-pituitary-thyroid axis, and the hypothalamic-neurohypophyseal axis) (4). Sensing can occur through cytokine-mediated central activation, or through ANS sensory fiber input, which can sense pathogens and tissue damage via receptors for DAMPs and PAMPs (2). In the case of hypothalamic-neurohypophyseal axis, the ANS baro- and chemoreceptors can also stimulate the release of vasopressin (4, 29).

Activation of the HPA axis results in cortisol release from the adrenal cortex, while activation of the SNS leads to a release of catecholamines from the adrenal medulla. The net effect of these pathways leads to increased glucose production and availability, which is an adaptive response to stress designed to enhance energy availability to vital organs (2). Cortisol can also enhance the response to catecholamines and has anti-inflammatory effects that can ultimately modulate ANS activation (34). Adrenal insufficiency, characterized by low cortisol levels, often develops in sepsis and is associated with poor outcomes (2). Observational studies have shown that adrenal insufficiency is associated with reduced HRV, and that response to hydrocortisone therapy is associated with recovery of normal HRV, further illustrating the close interactions between the ANS and the neuroendocrine response (23).

### Gastrointestinal function

The enteric nervous system branch of the ANS plays a key role in the modulation of gastrointestinal motility and digestion (6). The vagus nerve and the sacral parasympathetic nerves promote motility, sphincter relaxation, and digestive gland secretion (including salivary, gastric, pancreatic glands). On the other hand, sympathetic stimulation of α-adrenergic receptors decrease gastrointestinal motility and sphincter constriction (1). During critical illness, parasympathetic suppression or excessive sympathetic activity can lead to functional ileus, digestive dysfunction, and feeding intolerance (6).

The ANS appears to also have a direct effect on the gut microbiome. For example, microbes are capable of responding to neuroendocrine hormones. Specifically, catecholamines may induce microbial change to more pathogenic phenotypes, and these microbes may have exponential growth in response to host stress within hours of sensing higher catecholamine levels (35, 36). In addition, the afferent sensory fibers of the vagus nerve are activated by microbes in the gut and appear to distinguish between pathogenic and non-pathogenic organisms. Further research is needed to determine the role of this sensing in the cholinergic anti-inflammatory pathway activity and the immunomodulatory effects of gut bacteria (37).

### Thermoregulation

Core body temperature is tightly regulated by the CNS via behavioral and autonomic responses. The behavioral responses (e.g., putting on or removing clothing) are by far the most powerful strategies, but many critically ill patients lack the ability to implement behavioral responses and thus depend heavily on autonomic defenses such as sweating and pre-capillary vasodilation in response to heat, and arteriovenous (AV) shunt constriction and shivering in response to cold (8). The AV shunts are short connections between arterioles and veins that primarily exist in the distal extremities, and can carry about 10,000 times more flow than capillaries of the same length (8, 38). As such, these AV shunts, which are heavily innervated by sympathetic fibers, can significantly change the peripheral blood flow resistance. When open, lower resistance allows for significant flow to the extremities so that heat dissipates; closed they increase the resistance and reduce flow to the extremities, keeping the body-generated heat in the core (8, 38). Despite their limited distribution in the body, AV shunts have a profound effect on core temperature, and are the most commonly activated thermoregulatory defense in cool environments.

When hyperthermic, skin vasodilation can have significant impact on hemodynamics given that skin blood flow can increase up to the equivalent of 60% of the cardiac output (38). In homeostasis, autonomic responses work in concert, and sympathetic input to the heart will often match the cardiac output demands, but this may not be the case in some critical care situations.

## Measures of autonomic nervous system function in the intensive care unit

### General considerations

Potential confounders that could affect the measurement of ANS function in the pediatric intensive care unit include age, mechanical ventilation, neuromuscular blockade, the administration of catecholamines and sedatives, and pre-existing autonomic dysregulation. Some studies in critically ill adults suggest that mechanical ventilation and catecholamines do not affect HRV or baroreflex and chemoreflex sensitivity (13, 39). Sedation, on the other hand, appears to affect HRV and RRV mostly in patients with low organ dysfunction burden, but it does not appear to significantly affect HRV in more critically ill patients (13, 40, 41).

### Heart rate variability

Cardiovascular homeostasis requires continuously varying levels of sympathetic and parasympathetic inputs to the heart, resulting in a continuously changing heart rate, even during normal resting conditions (12). HRV is defined as the fluctuation in time between consecutive heart beats that is present in normal physiologic conditions (42). ANSD may result in an imbalance between sympathetic and parasympathetic inputs, usually resulting in a reduced HRV (12).

There are several examples of successful evaluations of real-time analysis of HRV in the intensive care unit (19, 22, 43–45). Nemati et al. recently described a real-time sepsis prediction tool for critically ill adult patients, which included, amongst multiple measures, heart rate entropy, a measure of HRV. This observational study showed that electronic health record data can predict sepsis 4 to 12 h earlier than SOFA scores or clinical suspicion of sepsis, with a areas under the receiver operating characteristic curve of 0.83–0.85 (44). Moorman et al. evaluated a real-time heart rate characteristic scoring tool for sepsis prediction in premature neonates, which was associated with a significantly decreased risk of mortality in a multi-center study, with a number needed to monitor of 48 neonates to prevent one death (19). These studies demonstrate the feasibility of incorporation of HRV evaluation into routine clinical care with the potential benefit of identifying disease trajectory and response to therapy in patients with sepsis.

### Respiratory rate variability

Similar to HRV, the ANS regulates variability in the respiratory rate with input from chemoreceptors and baroreceptors (46). Dysregulated RRV can be seen in many disease states. For example, altered RRV has been associated with extubation failure in adults (15, 16, 47). In addition, one study showed that lower RRV was associated with increased mortality in mechanically-ventilated patients, even when controlling for neuromuscular blockade (14). While there is some confounding of RRV by sedatives, measurement of this variability has been found to be reliable in adult ICUs (21, 40). Research studies are lacking in pediatrics and in the sepsis population, however, given the importance of autonomic control over breathing, it is likely that dysregulated RRV is common in sepsis.

### Baroreflex sensitivity

The baroreflex sensitivity measures the ability of the ANS to modulate the vagal and sympathetic tone with changes in blood pressure (30). Invasive methods like the phenylephrine bolus technique to evaluate lengthening of the heart beat interval after increasing the systolic blood pressure has been used in the clinical setting, but other non-invasive measures of baroreflex sensitivity, based on the effects of unprovoked fluctuations of blood pressures on heart rate, also exist (12). Decreased baroreflex sensitivity has been documented in critically ill adults with organ dysfunction and in animal models of sepsis (13, 48).

### Chemoreflex sensitivity

Stimulation of the peripheral and central chemoreceptors by decreases in arterial oxygen levels and/or pH or increases in arterial carbon dioxide produce an increase in respiratory rate and sympathetic tone. In the ICU, chemoreflex sensitivity can be calculated as the regression slope of arterial oxygen tension to heart rate interval duration (12). Decreased sensitivity has been associated with organ dysfunction in critically ill patients, including in those with sepsis (13).

## Autonomic nervous system dysfunction in pediatric sepsis: current state and future directions

As a key component of the host response to infection, dysregulation of the ANS response is likely an important contributing factor to organ dysfunction in patients with sepsis (18). ANSD, typically measured using HRV, is prevalent in pediatric sepsis and correlates with disease severity and trajectory (10, 49). Recognition of ANSD may lead to earlier diagnosis of sepsis, which could have therapeutic and prognostic implications for patients, including earlier initiation of therapies, shorter duration of organ dysfunction, and decreased mortality (44). For example, using bedside HRV monitoring as a predictive tool of sepsis in premature neonates has been associated with decreased risk of mortality (19, 50–52). The limited use of non-invasive monitoring of ANS function in the ICU is in part due to technological complexities, particularly in children, although the digitalization of the healthcare system is facilitating the implementation of the systems and algorithms necessary to do so (53, 54). In addition, HRV varies with patient factors such as age, gender, medications, and mechanical ventilation, which creates challenges for its use and interpretation at the bedside. Further research is necessary to determine the individual and additive effect of these factors on HRV and its usefulness as a predictor. In addition, novel measures of ANS function, such as continuous temperature monitoring, automated pupillometry, and non-invasive baroreflex and chemoreflex sensitivity have shown promise in the monitoring of ANSD in other populations and could potentially have application as effective biomarkers in pediatric sepsis, but further study is necessary. (12, 55–57).

Advanced understanding of ANSD pathophysiology in pediatric sepsis could also help identify potential therapeutic targets. Sympathetic over-activity, which is common in sepsis and other critical illnesses, can lead to tachyarrhythmias, diastolic dysfunction, inflammation, immune suppression, platelet aggregation, and gastrointestinal dysmotility (3, 5, 6, 27, 33). Additionally, excessive sympathetic outflow has been postulated to cause catecholamine-resistant vasoplegia via α-adrenergic receptor desensitization (29). Similarly, suppressed vagal tone has been associated with chronic inflammation and functional ileus, which can also hinder recovery from sepsis (5, 6). Whether modulating sympathetic and parasympathetic activity could lead to improved outcomes in pediatric sepsis remains to be studied. However, some human and animal studies show promise. For example, sympathetic suppression with α_2_ agonists like dexmedetomidine and ß_1_-blockade with esmolol has been associated with improvement in vasoplegia in shock states (29). Statins can reduce sympathetic outflow and preserve parasympathetic tone, and have been associated with reduced inflammation and lower likelihood of developing sepsis in adult patients (12, 58). And vagal nerve stimulation has been associated with reduced inflammation and improved gut and lung epithelial integrity in animal and human studies, although its role in sepsis has not been explored (5, 7, 59).

## Conclusion

The ANS plays a major role in maintaining homeostasis in response to infections and other types of stress. ANSD represents an imbalanced or maladaptive response and is prevalent in pediatric sepsis. Better understanding of ANSD in pediatric sepsis could have significant diagnostic, prognostic, and therapeutic implications.

## Author contributions

LS-P and CB designed the review. All four authors made substantial contributions to drafting and final approval of the manuscript.

### Conflict of interest statement

The authors declare that the research was conducted in the absence of any commercial or financial relationships that could be construed as a potential conflict of interest. The reviewer AI and handling Editor declared their shared affiliation.

## Abbreviations

*PAMPs, DAMPs*: pathogen- and damage-associated molecular patterns
*TLRs*: toll-like receptors
*CRH*: corticotropin-releasing hormone
*ACTH*: adrenocorticotropic hormone
*incr*.: increased.
